# Cytokinin-GLK regulatory module promotes the acquisition of photosynthetic activity and cell cycle re-entry in a green-flower mutant of *Narcissus tazetta* var. *chinensis*

**DOI:** 10.3389/fpls.2026.1831530

**Published:** 2026-05-20

**Authors:** Wen-Hui Zhang, Fu-Jian Zhang, Feng Gao, Zhen-Jun Li, Li-Juan Wang, Bo Wang, Ri-He Peng, Yong-Dong Deng, Quan-Hong Yao

**Affiliations:** 1 Biotechnology Research Institute of Shanghai Academy of Agricultural Sciences, Shanghai Key Laboratory of Agricultural Genetics and Breeding, Shanghai, China; 2 Key Laboratory for Safety Assessment (Environnment) of Agricultural Genetically Modified Organisms, Ministry of Agriculture and Rural Affairs, Shanghai, China; 3 Agricultural Technology Extension Center of Chongming District, Shanghai, China

**Keywords:** cytokinin, GLK transcription factor, heterotrophic-to-photoautotrophic transition, Narcissus, transcriptomics and metabolomics

## Abstract

Floral organs typically function as heterotrophic tissues, heavily reliant on imported photoassimilates for respiration and development. However, under specific genetic or environmental conditions, they can reacquire photosynthetic capabilities. Here, we identified a rare green-flower (GF) mutant in *Narcissus tazetta* var. *chinensis* that exhibits stable green coloration and leaf-like characteristics. Physiological assays demonstrated that this mutant undergoes a fundamental metabolic transition from a heterotrophic state to a photoautotrophic state, characterized by functional Photosystem II activity and positive net photosynthetic rates. Integrated transcriptomic and metabolomic analyses identified the ectopic accumulation of Cytokinins (specifically Zeatin) as the primary key promoter of this transition. High Cytokinin levels correlated strongly with the activation of *GOLDEN2-LIKE* (*NcGLK*) transcription factors, which coordinated chloroplast biogenesis, stomatal development, and cell cycle re-entry. We propose a “Cytokinin-GLK” regulatory module governing floral greening, providing new insights into floral organ plasticity and potential strategies for breeding long-lasting ornamental varieties.

## Graphical Abstract

**Figure:**
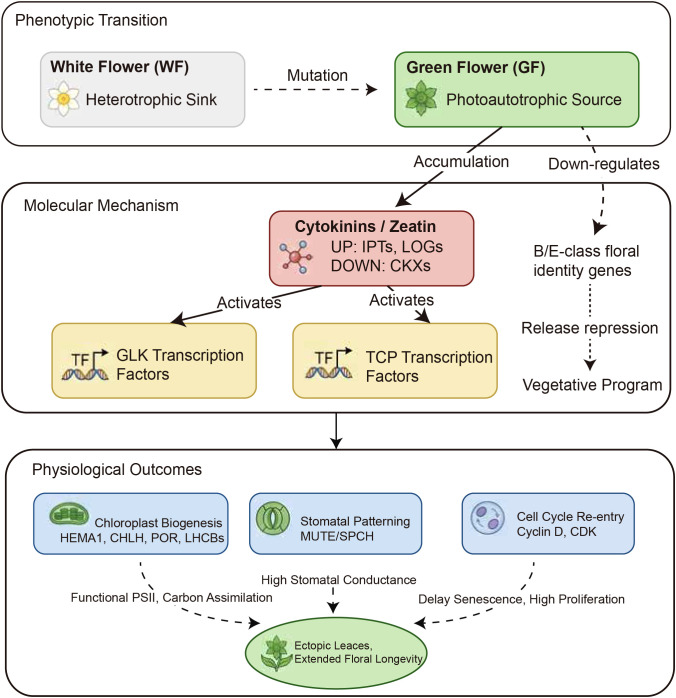

## 1 Introduction

Floral coloration is a critical evolutionary trait for angiosperms, serving primarily to attract pollinators for reproductive success (Grotewold, 2006). During floral development, the transition from green buds to colorful open flowers typically involves the disassembly of chloroplasts and their conversion into chromoplasts, accompanied by the degradation of chlorophylls and the accumulation of specialized pigments such as anthocyanins and carotenoids (Tanaka et al., 2008). Consequently, fully developed petals generally function as heterotrophic “sink” organs, relying on imported carbon skeletons from foliar “source” tissues to sustain their respiration and metabolism (Aschan and Pfanz, 2003). While green flowers occur naturally in some lineages or as mutants, they are relatively rare in nature because the retention of chlorophyll often renders the flower inconspicuous to pollinators (van der Kooi et al., 2019).

The formation of green flowers is often associated with the “stay-green” phenomenon or floral homeotic transformations (phyllody). Genetic studies have identified several key regulators governing this process. For instance, the *STAY-GREEN (SGR)* gene family encodes magnesium-dechelatases that are essential for chlorophyll degradation; loss-of-function mutations in *SGR* lead to the retention of green color in senescing leaves and floral tissues (Armstead et al., 2007; Sato et al., 2007). Furthermore, the *Golden2-like (GLK)* transcription factors act as master regulators of chloroplast biogenesis and photosynthesis-related gene expression. Ectopic expression of GLKs can induce chloroplast development in non-photosynthetic organs, including roots and petals (Susila et al., 2023; Zubo et al., 2018). Additionally, according to the classic ABCE model of floral development, mutations in B-class genes (e.g., *APETALA3* or *PISTILLATA*) can cause the homeotic transformation of petals into sepaloid or leaf-like structures, thereby reactivating the photosynthetic program (Coen and Meyerowitz, 1991). Furthermore, the maintenance of floral organ identity requires continuous genetic regulation to prevent “floral reversion”. This phenomenon occurs when floral meristems or developing organs lose their determinacy and revert to vegetative growth (Battey and Tooke, 2002). This process is tightly controlled by complex flowering pathways and hormonal crosstalk. When the genetic networks maintaining floral determinacy are compromised, floral organs can re-acquire leaf-like traits, including photosynthetic capacity (Hernández-Verdeja, 2026). However, while the genetic basis of pigment retention is partially understood, the systematic metabolic reprogramming and the physiological consequences of such “floral greening”—specifically the transition from a metabolic sink to a photosynthetic source—remain poorly characterized, especially in monocots with complex floral structures.

Narcissus (*Amaryllidaceae*) is a genus of economically important ornamentals characterized by a unique floral structure known as the corona (paracorolla), located between the tepals and stamens. In *Narcissus tazetta* var. *chinensis*, the wild-type flowers typically display white tepals and a yellow corona. Recently, we identified a rare spontaneous mutant exhibiting a stable green-flower phenotype, where both the tepals and the corona retain high chlorophyll levels and exhibit leaf-like morphological characteristics throughout anthesis. This mutant provides a unique opportunity to dissect the molecular and metabolic networks underlying floral organ identity and chloroplast maintenance (Cortleven et al., 2016).

In this study, we employed an integrated multi-omics approach combining transcriptomics and metabolomics, complemented by physiological assays, to investigate the mechanisms driving the green-flower phenotype in Narcissus. We hypothesized that this phenotype is not merely a defect in pigment degradation but represents a fundamental “sink-to-source” metabolic transition driven by hormonal signals. Our results reveal that the accumulation of cytokinins coordinates with GLK-mediated transcriptional networks to maintain functional chloroplasts, active photosynthesis, and cell division in the floral organs. These findings provide new insights into the plasticity of floral metabolism and the regulatory networks governing organ identity in plants.

## 2 Materials and methods

### 2.1 Plant materials and growth conditions

The WF and GF individuals of *Narcissus tazetta* var. *chinensis* were cultivated under natural field conditions at Shanghai. The soil type was a well-drained loam (pH ~6.5). Plants were grown under natural sunlight with an average daytime temperature of 20 °C, relative humidity ranging from 60-80%, and were subjected to standard field irrigation avoiding waterlogging. Both phenotypes were grown under identical soil, irrigation, and light conditions to minimize environmental variation.

For multi-omics and physiological analyses, floral tissues were sampled at the full-bloom stage (anthesis). The tepals and corona were dissected, and samples intended for RNA-seq and metabolomics were immediately frozen in liquid nitrogen and stored at -80 °C until extraction. Fresh tissues were used immediately for pigment quantification and photosynthetic measurements. Three biological replicates were collected for each phenotype.

### 2.2 Chlorophyll content measurement

Fresh floral tissues 0.1 g were homogenized in 10 mL of 80% acetone and incubated in the dark at room temperature until the tissue was completely bleached. The extract was centrifuged to remove debris. Absorbance of the supernatant was measured at 663 nm and 645 nm using a UV-visible spectrophotometer (UV-2700i). The concentrations of chlorophyll *a* (Chl *a*), chlorophyll *b* (Chl *b*), and total chlorophyll were calculated according to the standard equations (Gao et al., 2015):

Chl *a* (mg/g FW) = (12.7 × A663 - 2.69 × A645) × V/(1000 × W).
Chl *b* (mg/g FW) = (22.9 × A645 - 4.68 × A663) × V/(1000 × W).
Total Chl (mg/g FW) = Chl *a* + Chl *b*.

where V is the final volume of the extract (mL) and W is the fresh weight of the sample (g).

### 2.3 Physiological measurements of photosynthesis

#### 2.3.1 Chlorophyll fluorescence imaging

The maximum quantum yield of Photosystem II (*F_v_*/*F_m_*) was measured using a chlorophyll fluorescence imaging system Imaging-PAM, Walz, Germany. Before measurement, WF and GF plants were dark-adapted for 30 min to ensure reaction centers were fully open. *F_v_*/*F_m_* was calculated as (*F_m_* − *F_o_*)/*F_m_* (Brunello et al., 2024).

#### 2.3.2 Gas exchange measurements

Net photosynthetic rate (*P_n_*) and stomatal conductance (*g_s_*) were measured using a portable photosynthesis system LI-COR LI-6400XT on attached floral organs. Measurements were conducted between 9:00 and 11:00 AM under saturating light conditions (PPFD = 1000 μmol m^−2^ s^−1^), with a reference CO_2_ concentration of 400 μmol mol^−1^, a flow rate of 500 µmol s^−1^, and a leaf chamber fan speed of 10000 rpm, and a leaf chamber temperature of 25 °C (Gao et al., 2015).

### 2.4 Widely targeted metabolomics analysis

#### 2.4.1 Sample preparation and extraction

The frozen samples were ground into a fine powder using a mixer mill with liquid nitrogen. Approximately 100 mg of powder was extracted with 1.0 mL of 70% aqueous methanol. The mixture was vortexed, incubated at 4 °C overnight, and centrifuged. The supernatant was filtered through a 0.22 μm microporous membrane prior to LC-MS/MS analysis.

#### 2.4.2 Liquid chromatography and mass spectrometry conditions

Metabolite profiling was performed using a UPLC-MS/MS system (Shimadzu Nexera X2 coupled with Applied Biosystems 4500 QTRAP). Separation was achieved on an Agilent SB-C18 column. The mobile phases consisted of solvent A 0.1% formic acid in water and solvent B acetonitrile with 0.1% formic acid.

#### 2.4.3 Data analysis

Metabolites were identified based on a self-built database and public databases using retention time and fragmentation patterns. Quantification was performed using the Multiple Reaction Monitoring (MRM) mode. Principal Component Analysis (PCA) and Orthogonal Partial Least Squares Discriminant Analysis (OPLS-DA) were conducted to assess group clustering. Differentially accumulated metabolites (DAMs) were screened based on the criteria: Variable Importance in Projection (VIP) > 1 and |Log_2_Fold Change| ≥ 1. KEGG pathway enrichment analysis was performed to identify significantly altered metabolic pathways.

### 2.5 Transcriptomic sequencing and analysis

#### 2.5.1 RNA extraction and sequencing

Total RNA was extracted from the floral tissues using TRIzol Reagent following the manufacturer’s instructions. RNA integrity was assessed using the Agilent 2100 Bioanalyzer. Sequencing libraries were generated using the NEBNext^®^ Ultra™ RNA Library Prep Kit for Illumina^®^ (NEB, USA) and sequenced on an Illumina NovaSeq 6000 platform to generate paired-end reads.

#### 2.5.2 Data processing and DEG identification

Clean reads were obtained by removing low-quality reads and adapters. The high-quality reads were mapped to the *Narcissus* reference genome using HISAT2 (v2.1.0). On average, 85.5% of the clean reads successfully aligned to the reference genome, resulting in the identification of 45,230 expressed transcripts. Gene expression levels were calculated using FPKM (Fragments Per Kilobase of transcript per Million mapped reads) via featureCounts (v1.6.2). Differentially expressed genes (DEGs) between WF and GF were identified using the DESeq2 R package (v1.22.2) with thresholds of |Log_2_Fold Change| ≥ 1 and Adjusted *P*-value (FDR) < 0.05. Gene Ontology (GO) and KEGG enrichment analyses were performed to annotate biological functions.

### 2.6 Integrated transcriptome and metabolome analysis

To investigate the regulatory networks governing the GF phenotype, a Pearson Correlation Coefficient (PCC) matrix was constructed between the expression levels of DEGs and the abundance of DAMs. Gene-metabolite pairs with |PCC| > [0.8] and *P*-value < 0.05 were considered significantly correlated. The resulting regulatory networks, focusing on photosynthesis, cell cycle, and hormone signaling modules, were visualized using Cytoscape software (v3.8.0).

### 2.7 Statistical analysis

Statistical analyses were performed using R. Differences between the two groups (WF vs. GF) were evaluated using the Student’s *t*-test. Data are presented as mean ± standard deviation (*SD*). A *P*-value < 0.05 was considered statistically significant. All experiments, including omics analyses, physiological measurements, and qPCR, were performed with a minimum of three independent biological replicates.

## 3 Result

### 3.1 Identification and physiological characterization of a natural green-flowered mutant in *Narcissus*

**Figure 1:**
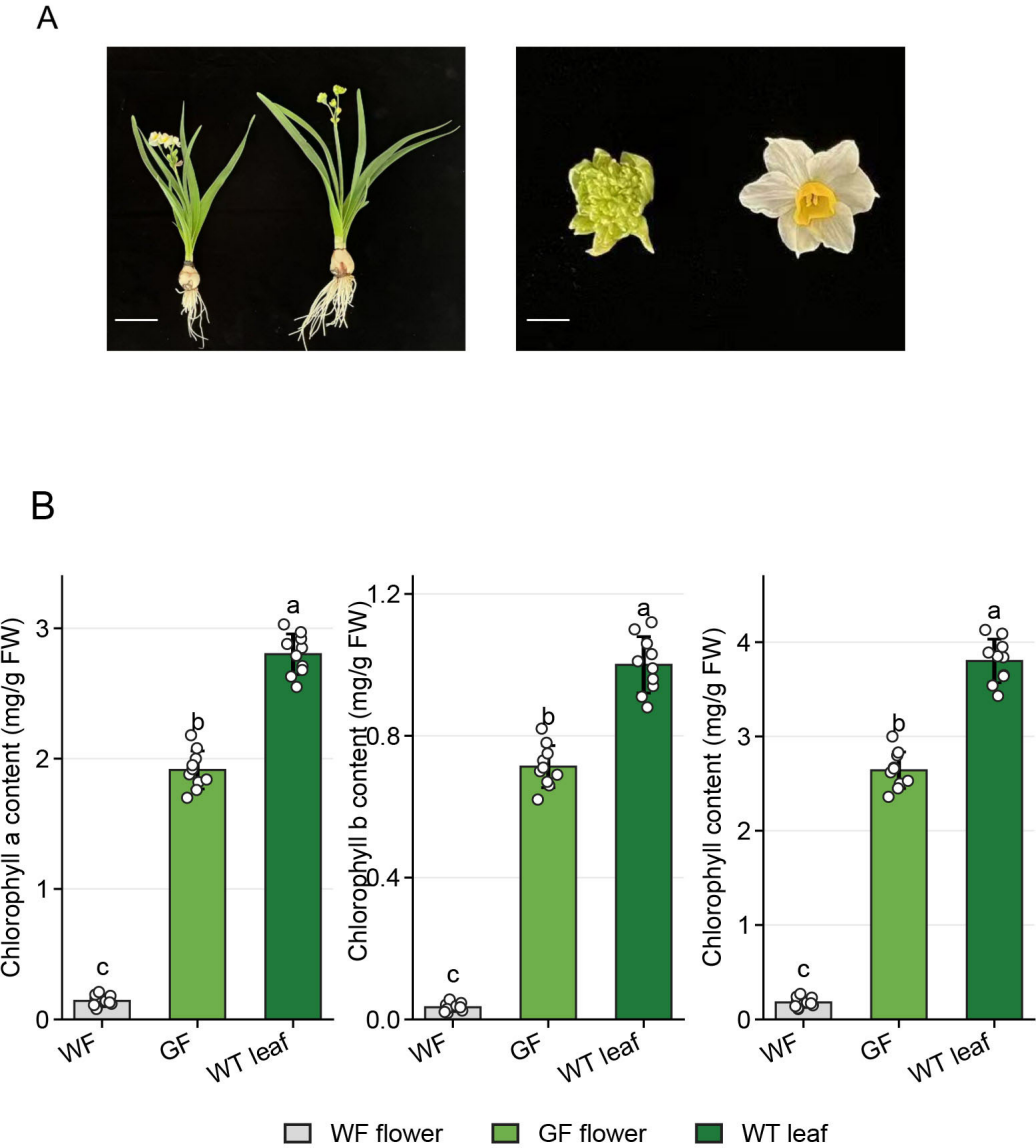
Phenotypic characterization and chlorophyll quantification of the wild-type flower, green-flower mutant, and wild-type leaf in Narcissus. **(A)** Morphological comparison between the wild-type flower (WF) and the green-flower mutant (GF) at the flowering stage. The left panel shows whole-plant architecture, and the right panel displays a close-up view of flowers. Scale bars = 5 cm (left) and 1 cm (right). **(B)** Quantification of photosynthetic pigments in WF floral tissues, GF floral tissues, and WT leaves. Chlorophyll a, chlorophyll b, and total chlorophyll contents were measured from fresh tissues. Data are presented as mean ± *SD*, and open circles represent individual biological replicates. Different letters indicate significant differences among groups (one-way *ANOVA* followed by Tukey’s test, *P* < 0.05).

Flower color is a critical trait for ornamental value and pollinator attraction. In a natural population survey of *Narcissus tazetta* var. *chinensis* (Chinese Sacred Lily), we identified a rare spontaneous mutant exhibiting a stable green phenotype. Unlike the WF, which display characteristic white tepals and yellow coronas at anthesis, the GF mutant showed consistent green coloration across both the tepals and the corona structure (**Figure 1A**). Notably, the inner reproductive whorls (stamens and pistil) of the GF mutant developed normally without obvious homeotic transformations, indicating that the phenotypic alteration is primarily restricted to the perianth.

To determine whether this phenotypic alteration was driven by the ectopic accumulation of photosynthetic pigments, we quantified the chlorophyll content in fresh WF floral tissues, GF floral tissues, and WT leaves at the full-bloom stage. As expected, chlorophyll levels in WF petals were negligible. In sharp contrast, GF tissues accumulated substantial amounts of chlorophyll a and chlorophyll b, resulting in a markedly higher total chlorophyll content than WF tissues (**Figure 1B**). WT leaves, used as a positive photosynthetic control, showed the highest pigment content among the three sample types. Although the chlorophyll levels in GF flowers remained lower than those in WT leaves, their strong enrichment relative to WF flowers supports the acquisition of a leaf-like photosynthetic pigment profile.

### 3.2 Metabolomic reprogramming reveals a sink-to-Source transition and hormone-Driven chloroplast maintenance in GF

**Figure 2:**
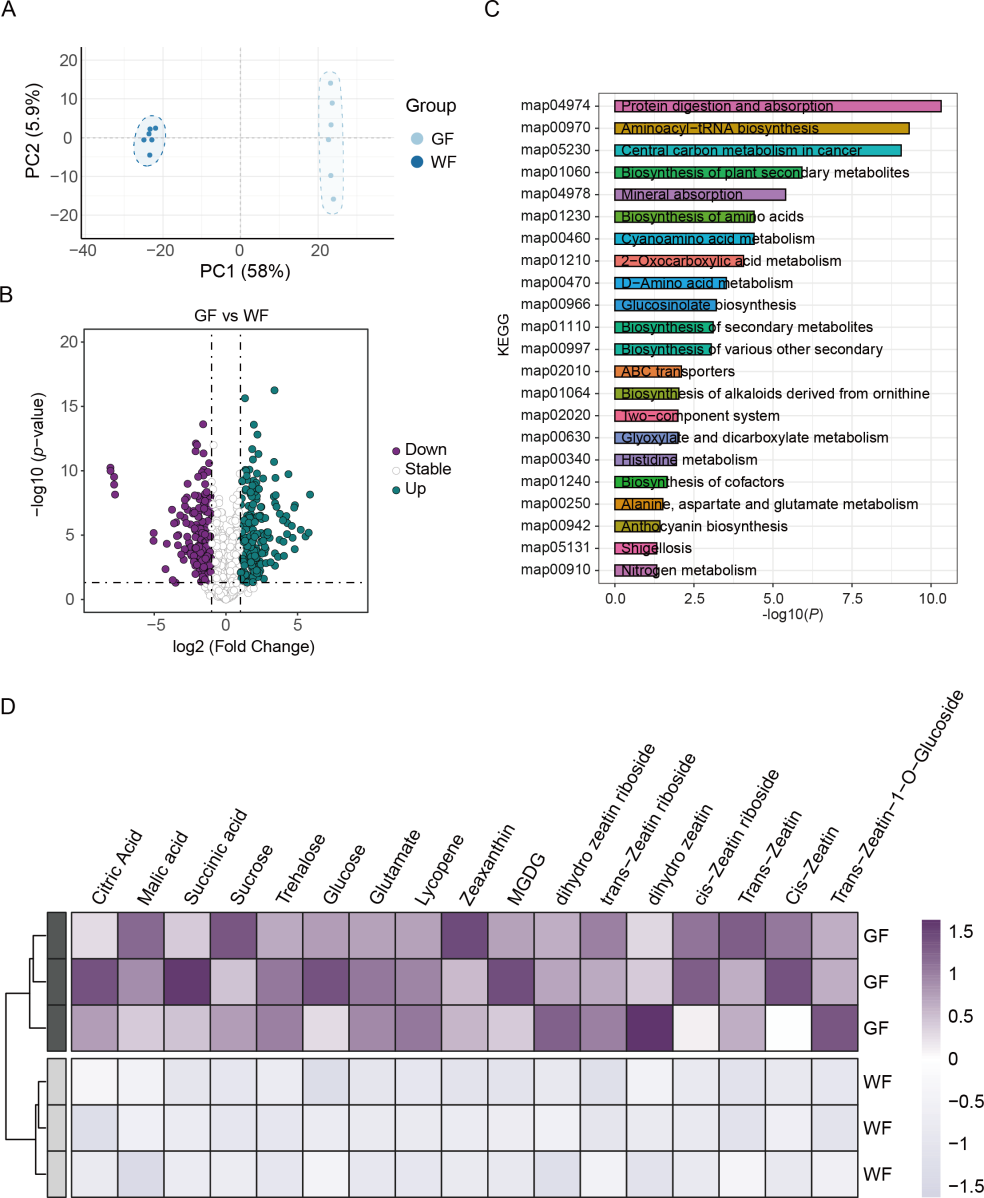
Metabolomic reprogramming between wild-type (WF) and green-flower mutant (GF) floral tissues. **(A)** Principal Component Analysis (PCA) score plot derived from the metabolomic profiles of WF and GF. The x-axis and y-axis represent the first principal component (PC1, explaining 58% of the variance) and the second principal component (PC2, explaining 5.9% of the variance), respectively. The distinct clustering indicates significant metabolic differences between the two phenotypes. **(B)** Volcano plot illustrating the differentially accumulated metabolites (DAMs) in GF compared to WF. Each dot represents a metabolite. Green dots indicate significantly up-regulated metabolites, purple dots indicate significantly down-regulated metabolites, and gray open circles indicate metabolites with no significant difference. Differentially accumulated metabolites (DAMs) were defined based on the criteria: *p* < 0.05 and |Log2Fold Change| ≥ 1. **(C)** KEGG pathway enrichment analysis of the identified DAMs. The bar chart shows the top enriched metabolic pathways. The x-axis represents the significance of enrichment (–log10 *P*-value), and the y-axis lists the pathway names. **(D)** Heatmap illustrating the relative abundance of selected differentially accumulated metabolites (DAMs) in GF and WF. The columns represent representative metabolite. The rows represent three biological replicates for the GF and WF phenotypes. The color scale indicates the normalized relative abundance of each metabolite, ranging from light gray (lower accumulation) to dark purple (higher accumulation).

To delineate the metabolic landscape underlying the GF phenotype, we performed a widely targeted metabolomic analysis on the tepals and corona of WF and GF. Principal component analysis (PCA) revealed a clear separation between the two phenotypes (**Figure 2A**). Furthermore, compared with WF, a total of 202 up-regulated and 181 down-regulated differentially accumulated metabolites were detected in GF (**Figure 2B**). These results indicate that the transition of flower color led to floral metabolic reprogramming.

KEGG pathway enrichment analysis highlighted significant alterations in “Porphyrin and chlorophyll metabolism,” “Carbon metabolism,” “Biosynthesis of amino acids,” and “Plant hormone signal transduction” (**Figure 2C**). Specifically, we observed a coordinated upregulation of metabolites associated with carbon assimilation and energy production. Key intermediates of the TCA cycle (e.g., citric acid, malic acid, succinic acid) and soluble sugars (e.g., sucrose, trehalose, glucose) were significantly enriched in GF compared to WF (**Figure 2D**). This accumulation pattern indicates enhanced photosynthetic and carbon fixation potentials in GF floral tissues. Furthermore, the metabolic flux appeared to be redirected toward chlorophyll and pigment biosynthesis. Glutamate, the fundamental precursor for chlorophyll biosynthesis, was significantly upregulated in GF. Interestingly, we also detected elevated levels of accessory pigments, including lycopene and zeaxanthin, as well as thylakoid membrane lipids (e.g., MGDG), which are essential for the assembly and photoprotection of functional photosystems (**Figure 2D**).Crucially, the hormonal profile of GF showed a striking accumulation of cytokinins, particularly Zeatin and its derivatives (**Figure 2D**). Since cytokinins are known to promote chloroplast differentiation and delay senescence, their accumulation correlates well with the “stay-green” phenotype. In summary, our metabolomic data demonstrate that the GF phenotype is driven by a comprehensive metabolic reprogramming centered on chloroplast functionalization. Concurrently, the coordinated upregulation of cytokinins, photosynthetic pigments, and carbon assimilation intermediates endows the GF floral tissues with an enhanced potential for photosynthetic autonomy.

### 3.3 Transcriptomic profiling uncovers a coordination of photosynthetic activation and cell cycle re-entry in GF

**Figure 3:**
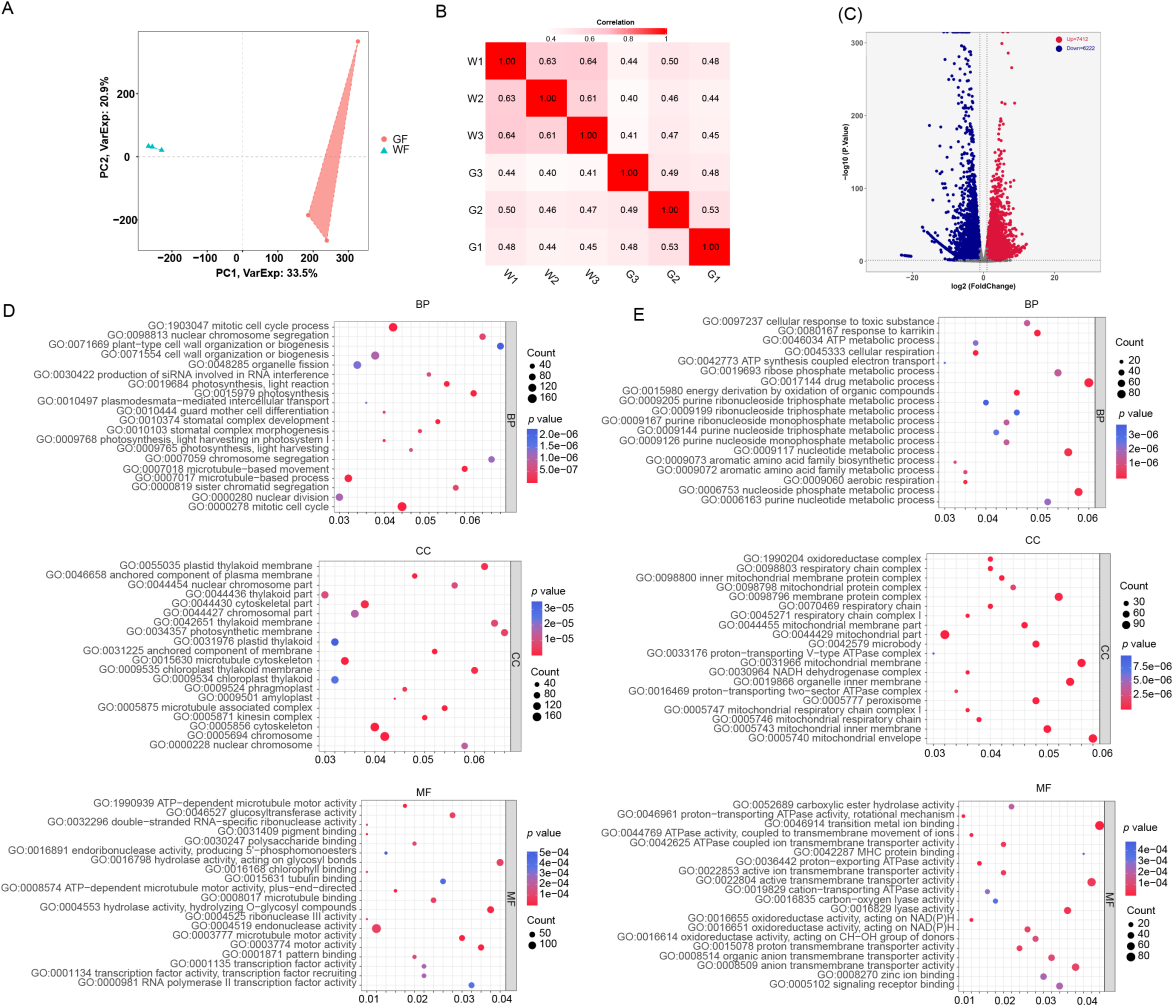
Transcriptomic profiling and functional enrichment analysis of differentially expressed genes (DEGs) between wild-type (WF) and green-flower mutant (GF). **(A)** PCA of the RNA-seq datasets.PC1 and PC2 explain 33.5% and 20.9% of the total variance, respectively. **(B)** Pearson correlation heatmap showing the reproducibility among biological replicates. The color scale (0.4-1.0) represents the correlation coefficient (*R*), with darker red indicating higher correlation. **(C)** Volcano plot visualizing the distribution of DEGs. The x-axis represents the log2 fold change, and the y-axis represents the statistical significance (-log10 *P*-value). Red dots denote significantly up-regulated genes in GF, while blue dots denote significantly down-regulated genes. Differentially expressed genes (DEGs) were identified with thresholds of |Log2Fold Change| ≥ 1 and *p* < 0.05. **(D)** Gene Ontology (GO) enrichment analysis of the up-regulated genes in GF. The bubble plots display the top enriched terms in Biological Process (BP), Cellular Component (CC), and Molecular Function (MF) categories. **(E)** Gene Ontology (GO) enrichment analysis of the down-regulated genes in GF. The terms are enriched in mitochondrial functions and cellular respiration. For **(D, E)**, the dot size represents the number of genes (Count), and the color gradient represents the significance (*P*-value).

To dissect the molecular regulatory network driving the floral greening, we performed comparative transcriptomic analysis (RNA-seq) on the floral tissues of WF and GF (**Figures 3A, B**). We identified a total of 13634 differentially expressed genes (DEGs), comprising 7412 up-regulated and 6222 down-regulated genes in the GF mutant (**Figure 3C**). In addition, the accuracy of the differentially expressed genes was verified by qPCR (**Supplementary Figure 5**). Gene Ontology (GO) enrichment analysis revealed a fundamental transcriptomic reprogramming that orchestrates the phenotypic transition.

Consistent with the metabolomic and physiological data, the up-regulated genes in GF were predominantly enriched in pathways related to chloroplast biogenesis and photosynthesis. Terms such as “photosynthesis,” “chlorophyll binding,” and “thylakoid part” were highly overrepresented (**Figure 3D**). This expression profile suggests that the nuclear-encoded photosynthetic machinery is actively transcribed, potentially facilitating the development of functional chloroplasts in the floral organs.

Strikingly, beyond the photosynthetic machinery, we observed a massive activation of pathways associated with cell proliferation and tissue patterning. GO terms including “mitotic cell cycle,” “nuclear division,” “microtubule-based process,” and “phragmoplast” were significantly enriched among the up-regulated genes (**Figure 3D**). This suggests that unlike the determinate, fully differentiated cells in WF petals, the GF tissues retain or re-acquire a high proliferative potential. In contrast, the down-regulated genes were heavily enriched in “cellular respiration,” “mitochondrial part,” and “ATP generation” pathways (**Figure 3E**). This reduction in expression seems to align with a metabolic transition—from a heterotrophic state reliant on high respiration rates to derive energy from imported sugars, to a more autotrophic, source-like status.

A true transition from a sink to a source tissue involves not only photosynthetic activation but also shifts in sugar allocation and senescence progression. Therefore, we evaluated the expression profiles of known marker genes for these processes. We found that several sugar transporter genes, including Sucrose Transporters *(NcSUTs*) and Sugars Will Eventually be Exported Transporters (*NcSWEETs*), were significantly up-regulated in the GF mutant, indicating an enhanced capacity for phloem loading and sugar export. Concurrently, Senescence-Associated Genes (*NcSAGs*, such as *SAG12* and *SAG29* homologs), which are typically expressed in mature wild-type petals, were dramatically down-regulated in GF tissues (**Supplementary Figure 1**).

Given the leaf-like characteristics of the GF perianth, we further investigated the expression of classic ABCE floral organ identity genes. Our RNA-seq data revealed that while C-class genes (responsible for reproductive organ identity) showed stable expression, transcript levels of specific B-class genes (*APETALA3* and *PISTILLATA* homologs) and E-class genes (*SEPALLATA* homologs) were significantly down-regulated in the GF mutant compared to WF. This partial suppression of floral organ identity genes aligns with the acquisition of vegetative traits in the perianth (**Supplementary Figure 2**).

### 3.4 Integrated transcriptomic and metabolomic analysis reveals a cytokinin-GLK regulatory module driving floral greening

To elucidate the regulatory architecture linking gene expression changes to metabolic reprogramming, we performed a conjoint analysis of the transcriptome and metabolome datasets. A Pearson correlation coefficient (PCC) matrix was constructed to identify strong correlations between differentially expressed genes (DEGs) and differentially accumulated metabolites (DAMs).

**Figure 4:**
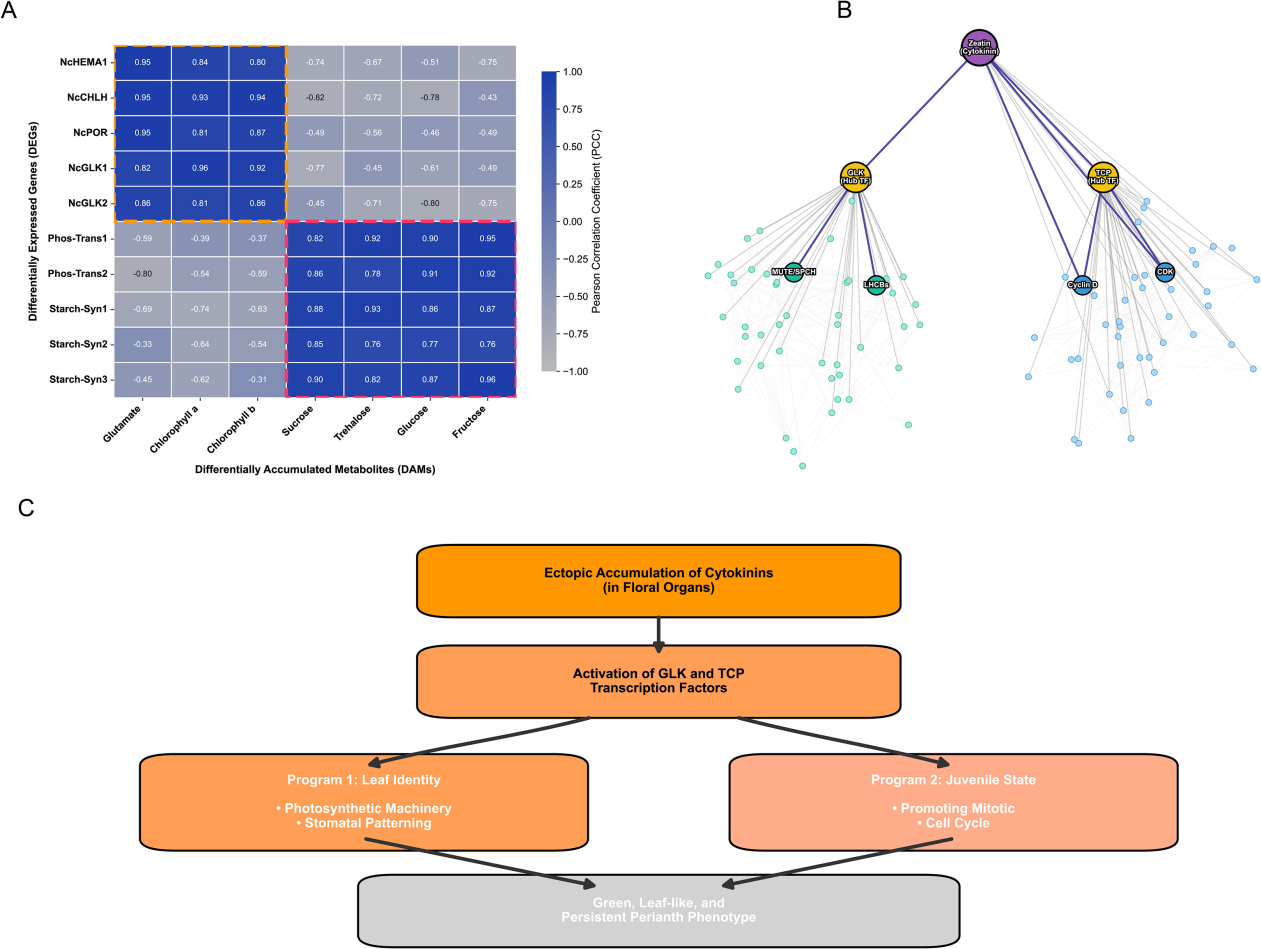
Integrated transcriptomic and metabolomic analysis reveals the regulatory network underlying the green and persistent perianth phenotype. **(A)** Pearson correlation coefficient (PCC) heatmap of key differentially expressed genes (DEGs) and differentially accumulated metabolites (DAMs). The orange and red dashed boxes highlight strong positive correlations within the chlorophyll/tetrapyrrole biosynthesis module and the soluble sugar/starch synthesis module, respectively. Dark blue indicates strong positive correlations, while gray indicates negative correlations. **(B)** Hub transcription factor (TF) co-expression network. The hierarchical network illustrates the regulatory signaling cascade from cytokinin (Zeatin, purple node) to the central hub TFs (GLK and TCP, yellow nodes), which subsequently regulate target genes associated with photosynthesis/stomatal development (green nodes) and the cell cycle (blue nodes). Dark blue edges indicate strong regulatory connections, while lighter edges represent module co-expression backgrounds. **(C)** A proposed mechanistic model. The ectopic accumulation of cytokinins in floral organs acts as the primary signal to activate GLK and TCP transcription factors. These regulators orchestrate a dual biological program: (1) conferring leaf identity (photosynthetic machinery and stomatal patterning) and (2) sustaining a juvenile state (promoting the mitotic cell cycle). Together, these programs result in the characteristic green, leaf-like, and persistent perianth phenotype.

The analysis revealed an interconnected “Gene-Metabolite Regulatory Network” (**Figure 4A**). We observed that the accumulation of Chlorophylls and Glutamate (the chlorophyll precursor) showed a positive correlation with the expression levels of key tetrapyrrole biosynthesis genes, including *NcHEMA1* (Glutamyl-tRNA reductase), *NcCHLH* (Magnesium-chelatase), and *NcPOR* (Protochlorophyllide oxidoreductase). Concurrently, the elevated levels of soluble sugars (Sucrose, Trehalose) were positively correlated with the upregulation of phosphate translocators and starch synthesis genes, confirming the synchronization of the photosynthetic gene network with carbon assimilation products.

Crucially, to understand the upstream regulators orchestrating this transformation, we focused on transcription factors (TFs) co-expressed with the “Photosynthesis” and “Cell Cycle” modules. We identified specific members of the GLK (Golden2-like) and TCP transcription factor families as central “hub” nodes in the network (**Figure 4B**). These TFs exhibited expression patterns parallel to the accumulation of Zeatin (cytokinin). Specifically, the *NcGLK* transcripts were strongly correlated with both light-harvesting complex genes (*LHCBs*) and stomatal development genes (*MUTE/SPCH* homologs) (**Supplementary Table 1**). Furthermore, the Cytokinin-Zeatin content showed a significant positive correlation with cell cycle regulators (e.g., *Cyclin D*, *CDK*), suggesting that the high cytokinin levels in GF may act as the primary signal to trigger cell division and prevent chloroplast senescence. Furthermore, the *NcGLK* transcripts were dramatically induced in the green-flower mutant, shifting from nearly undetectable levels in the wild-type (WF) to robust expression in GF. Recognizing that chloroplast biogenesis is often controlled by redundant or cooperative regulatory networks, we expanded our transcription factor profiling. This analysis revealed the concomitant induction of other well-known chloroplast-related master regulators, particularly members of the GATA family (e.g., homologs of *GNC* and *CGA1*) and the *MYB* transcription factor family (**Supplementary Figure 4**).

To understand the molecular basis of the massive cytokinin accumulation observed in our metabolomic data, we further investigated the expression profiles of common cytokinin biosynthesis and degradation genes. A targeted heatmap (**Supplementary Figure 3**) revealed a coordinated transcriptomic shift favoring CK accumulation in the GF mutant. Specifically, key biosynthesis genes, including Isopentenyltransferases (*NcIPTs*) and LONELY GUY (*NcLOGs*), exhibited up-regulation. Conversely, transcripts encoding Cytokinin Oxidase/Dehydrogenase (*NcCKXs*), which are responsible for CK degradation, were markedly down-regulated. These results indicate that the GF phenotype may be driven by endogenous *de novo* cytokinin biosynthesis, rather than merely a blockade of the degradation process.

Based on these integrated data, we propose a model (**Figure 4C**) wherein the ectopic accumulation of Cytokinins in the floral organs activates *GLK* and *TCP* transcription factors. These regulators subsequently trigger a dual program: (1) conferring leaf identity by activating the photosynthetic machinery and stomatal patterning, and (2) sustaining a juvenile state by promoting the mitotic cell cycle, ultimately resulting in the green, leaf-like, and persistent perianth phenotype.

### 3.5 Physiological validation confirms that green flowers function as active photosynthetic organs

To definitively verify whether the reprogrammed transcriptome and metabolome translate into functional photosynthetic activity, we conducted *in vivo* physiological measurements on the floral tissues of WF and GF.

**Figure 5:**
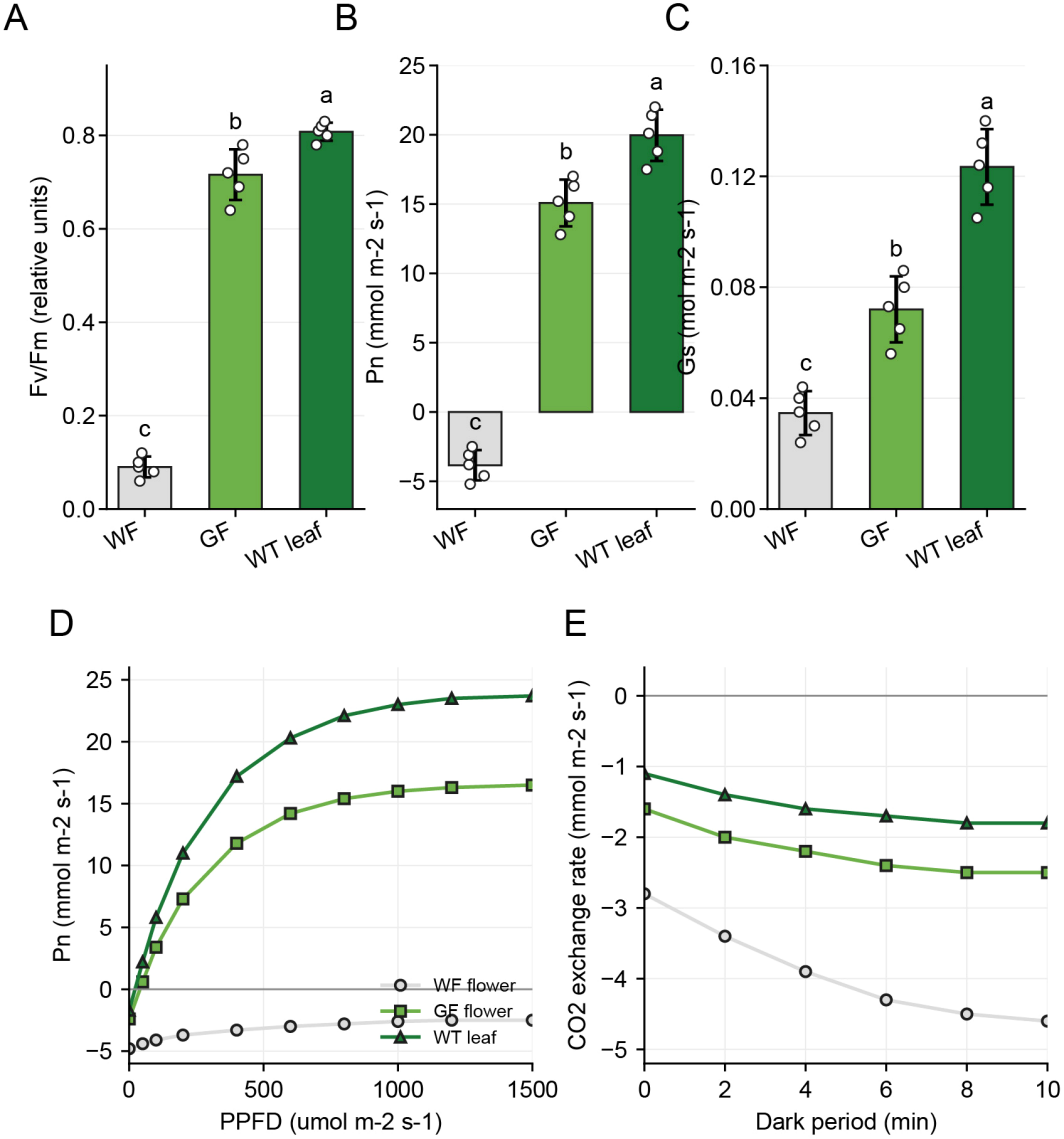
Physiological validation of leaf-like photosynthetic activity in the green-flower mutant using wild-type leaves as a positive control. **(A)** Maximum quantum yield of Photosystem II (*Fv/Fm*) in WF flowers, GF flowers, and WT leaves after dark adaptation. **(B)** Net photosynthetic rate (Pn) under saturating light conditions. WF flowers displayed negative values, indicating respiration-dominated CO2 release, whereas GF flowers and WT leaves displayed positive carbon assimilation. **(C)** Stomatal conductance (gs) in WF flowers, GF flowers, and WT leaves. **(D)** Light-response curves of Pn under increasing photosynthetic photon flux density (PPFD). **(E)** Dark respiration curves measured as CO_2_ exchange rates during the dark period. Data in **(A–C)** are presented as mean ± *SD*, and open circles represent individual biological replicates. Different letters indicate significant differences among groups (one-way *ANOVA* followed by Tukey’s test, *P* < 0.05).

First, we assessed the photochemical efficiency of Photosystem II (PSII) using chlorophyll fluorescence imaging with WT leaves included as a positive control. In WF tepals, the maximum quantum yield of PSII (*Fv/Fm*) was negligible, reflecting the absence of functional reaction centers. In striking contrast, GF tissues exhibited high *Fv/Fm* values approaching those of WT leaves (**Figure 5A**). This indicates that the chloroplasts assembled in the green flowers possess functional light-harvesting complexes and PSII reaction centers capable of efficient electron transport.

Furthermore, we measured gas-exchange parameters to evaluate net carbon assimilation. As expected for heterotrophic sink organs, WF flowers displayed negative Pn values, indicating that respiration (CO_2_ release) dominated their metabolism. However, GF flowers showed positive net photosynthetic rates under saturating light conditions, whereas WT leaves displayed the highest Pn values (**Figure 5B**). Light-response curves further showed that GF flowers responded positively to increasing PPFD and reached a clear photosynthetic plateau, although their maximum assimilation capacity remained lower than that of WT leaves (**Figure 5D**). This transition from net respiration to net carbon fixation substantiates the source-sink inversion hypothesis derived from our multi-omics data.

Consistent with the upregulation of stomatal development genes observed in the transcriptome, GF tissues also exhibited significantly higher stomatal conductance (gs) than WF flowers, while WT leaves showed the highest gs values (**Figure 5C**). Dark respiration assays showed that WF flowers maintained stronger net CO_2_ release in darkness, whereas GF flowers displayed lower respiratory CO_2_ release and were closer to WT leaves (**Figure 5E**). Together, the coordination of functional PSII, positive light-dependent CO_2_ assimilation, and enhanced stomatal conductance confirms that the green flowers have acquired substantial leaf-like photosynthetic capacity, while WT leaves define the upper physiological benchmark for a fully photosynthetic organ.

## 4 Discussion

### 4.1 Metabolic reprogramming drives a functional “sink-to-source” transition in the green-flower mutant

In this study, we characterized a rare green-flower (GF) mutant in *Narcissus tazetta* var. *chinensis*. Unlike typical “stay-green” mutants that result from defects in chlorophyll degradation pathways (e.g., *SGR* mutations) (Sato et al., 2007), our multi-omics data suggest that the GF phenotype represents an active maintenance of chloroplast biogenesis and a potential shift in organ identity. As introduced, floral organ identity is strictly governed by the ABCE model. The down-regulation of B- and E-class genes observed in the GF mutant suggests a partial loss of definitive petal identity. In typical floral development, B-class genes not only specify petal identity but also actively repress photosynthetic development to ensure the formation of a heterotrophic “sink” organ. The reduction in B/E-class gene expression in the GF mutant likely acts synergistically with the Cytokinin-GLK module, releasing the repression of the vegetative program and allowing the ectopic assembly of the photosynthetic apparatus (Wei et al., 2025). A particularly notable observation is the metabolic inversion from a heterotrophic “sink” to an autotrophic “source.” In wild-type (WF) flowers, petals rely on imported carbon for respiration, a process energetically costly for the plant. Conversely, the GF mutant exhibited upregulated TCA cycle intermediates, accumulated soluble sugars, and, most critically, a positive net photosynthetic rate (*P_n_*) coupled with functional PSII activity (*F_v_*/*F_m_* ~0.75). This physiological evidence, supported by the transcriptomic upregulation of carbon fixation enzymes, suggests that the GF floral organs may have developed the capacity for self-sustained carbon assimilation. Consequently, they could potentially function akin to ectopic leaves, possibly supplementing the plant’s overall carbon budget.

### 4.2 The cytokinin-GLK module as a master regulator of floral greening

Our integrated network analysis highlighted a pivotal role for Cytokinins (CKs) in coordinating this phenotypic transition. The massive accumulation of Zeatin in GF tissues, strongly correlated with the expression of *GOLDEN2-LIKE* (*NcGLK*) transcription factors, proposes a hierarchical regulatory model. In *Arabidopsis* and rice, GLK factors are known master regulators of nuclear-encoded photosynthetic gene (Waters et al., 2009). However, their regulation in non-foliar organs is less understood. Our data suggest that in *Narcissus*, ectopic high levels of Cytokinins likely act as the upstream signal to activate *NcGLK* expression, which subsequently triggers the expression of *LHCBs* and chlorophyll biosynthesis genes (*HEMA1*, *CHLH*, *POR*). This aligns with previous findings that Cytokinins can induce chloroplast differentiation in roots and calli (Kobayashi et al., 2012), but our study demonstrates this distinct regulatory module within the complex context of floral organ development.

### 4.3 Implications for floral longevity and breeding

The “greening” of floral organs has significant implications for flower longevity. Traditional petals are metabolically expensive and undergo rapid senescence once pollination occurs or energy reserves are depleted (Susila et al., 2023). In contrast, the autotrophic nature of the GF mutant allows it to generate its own energy. Supported by our transcriptomic data showing the down-regulation of senescence-associated genes (*SAGs*) and the up-regulation of sugar exporters (*SUTs/SWEETs*), the GF mutant not only delays developmentally programmed cell death but also potentially exports surplus photoassimilates. This trait is highly desirable in ornamental breeding. Understanding the *Cytokinin-GLK-Photosynthesis* regulatory axis provides potential genetic targets for engineering “everlasting” flowers that can maintain turgor and visual appeal for extended periods by subsidizing their own respiratory costs.

### 4.4 Limitations and future perspectives

While our integrated multi-omics and exogenous hormone treatments strongly suggest a Cytokinin-GLK regulatory axis, we acknowledge that much of the network remains predictive. Narcissus is a recalcitrant monocot lacking a robust *in vivo* genetic transformation or Virus-Induced Gene Silencing (VIGS) system, which currently limits direct functional validation in the native host. Future studies should focus on establishing transient assays or utilizing heterologous expression systems (e.g., in Arabidopsis) to validate the homeologs of *NcGLK*. Furthermore, molecular binding assays such as Chromatin Immunoprecipitation (ChIP), Electrophoretic Mobility Shift Assays (EMSA), and dual-luciferase reporter systems are required to definitively identify the direct downstream cis-elements bound by *NcGLK* during floral greening.

## 5 Conclusion

In conclusion, our study demonstrates that the green-flower mutant in *Narcissus* is not merely a consequence of defective pigment degradation but represents a profound physiological transition from a heterotrophic sink to a functional photoautotrophic source. Through integrated multi-omics and physiological validations, we identified a critical cytokinin-GLK regulatory module. The ectopic accumulation of cytokinins (zeatin) acts as an upstream signal to activate GLK transcription factors, which synergistically orchestrate chloroplast biogenesis, activate the photosynthetic apparatus, and promote cell cycle re-entry in floral organs. These findings provide novel molecular insights into the plasticity of floral organ identity and offer valuable genetic targets for breeding ornamental varieties with enhanced longevity and unique aesthetic traits.

## Data Availability

The original contributions presented in the study are included in the article/**Supplementary Material**. Further inquiries can be directed to the corresponding authors.

## Supplementary material

Supplementary Figure 1 Expression patterns of sugar transport and senescence-associated genes in WF and GF. The heatmap displays the differential expression of sucrose transporter genes (*NcSUTs*), sugar transporter genes (*NcSWEETs*), and senescence-associated genes *(NcSAGs*) between WF and GF samples. The color scale ranging from gray (−1.5) to brown (1.5) represents the *Z*-score normalized relative expression levels of the genes. Each column represents a biological replicate (*n* = 3).

Supplementary Figure 2 Expression patterns of floral organ identity genes in WF and GF. The heatmap illustrates the expression profiles of MADS-box family genes involved in floral organ development (including *NcAP3, NcPI, NcSEP1/2/3/4*, and *NcAG*) in WF and GF samples. The color scale from gray (−1.5) to brown (1.5) indicates the *Z*-score normalized relative expression levels. Each column represents a biological replicate (*n* = 3).

Supplementary Figure 3 Expression patterns of cytokinin metabolism-related genes in WF and GF. The heatmap analyzes the expression levels of genes involved in cytokinin biosynthesis (*NcIPTs, NcLOGs*) and degradation (*NcCKX*) in WF and GF. Biosynthesis-related genes are highly expressed in GF, whereas the degradation gene is highly expressed in WF. The color scale represents normalized expression levels (gray for low expression, brown for high expression). Each column represents a biological replicate (*n* = 3).

Supplementary Figure 4 Expression analysis of transcription factors related to chloroplast development and photosynthesis in WF and GF. Bar charts show the transcript abundance (represented by TPM values) of four transcription factors: *NcGLK*, *NcGNC*, *NcCGA1*, and *NcMYB12* in WF and GF. Error bars represent the standard deviation of three biological replicates (Mean ± *SD*), and circles represent individual data points. Asterisks indicate high statistical significance between the groups (Student’s *t*-test, ****p* < 0.001).

Supplementary Figure 5 Relative expression levels of representative genes involved in photosynthesis/chlorophyll biosynthesis (*NcLHCB, NcHEMA1*, and *NcCHLH*), the GLK regulatory module (*NcGLK*), cell-cycle progression (*NcCYCD3* and *NcCDKB*), and cytokinin metabolism *(NcIPT* and *NcCKX*) in wild-type flowers (WF) and green-flower mutant tissues (GF). Expression levels were calculated using the 2−ΔΔCT method and normalized to WF. Bars represent means ± *SD* of three biological replicates, and individual points indicate replicate values. Statistical significance was determined using a two-tailed Student’s *t*-test; ***P* < 0.01, ****P* < 0.001.

Supplementary Table 1 Putative downstream target genes strongly co-expressed with the *NcGLK* transcription factor in the floral greening regulatory network.

## References

[B1] Armstead I. Donnison I. Aubry S. Harper J. Hörtensteiner S. James C. . (2007). Cross-species identification of Mendel's I locus. Sci. (New York N.Y.) 315, 73. doi: 10.1126/science.1132912. PMID: 17204643

[B2] Aschan G. Pfanz H. (2003). Non-foliar photosynthesis – a strategy of additional carbon acquisition. Flora - Morphol Distrib Funct. Ecol. Plants 198, 81–97. doi: 10.1078/0367-2530-00080

[B3] Battey N. H. Tooke F. (2002). Molecular control and variation in the floral transition. Curr. Opin. Plant Biol. 5, 62–68. doi: 10.1016/s1369-5266(01)00229-1. PMID: 11788310

[B4] Brunello L. Polverini E. Lauria G. Landi M. Guidi L. Loreti E. . (2024). Root photosynthesis prevents hypoxia in the epiphytic orchid Phalaenopsis. Funct. Plant Biol. 51, FP23227. doi: 10.1071/fp23227. PMID: 38442921

[B5] Coen E. S. Meyerowitz E. M. (1991). The war of the whorls: genetic interactions controlling flower development. Nature 353, 31–37. doi: 10.1038/353031a0. PMID: 1715520

[B6] Cortleven A. Marg I. Yamburenko M. V. Schlicke H. Hill K. Grimm B. . (2016). Cytokinin regulates the etioplast-chloroplast transition through the two-component signaling system and activation of chloroplast-related genes. Plant Physiol. 172, 464–478. doi: 10.1104/pp.16.00640. PMID: 27388681 PMC5074628

[B7] Gao Y. Jiang W. Dai Y. Xiao N. Zhang C. Li H. . (2015). A maize phytochrome-interacting factor 3 improves drought and salt stress tolerance in rice. Plant Mol. Biol. 87, 413–428. doi: 10.1007/s11103-015-0288-z. PMID: 25636202

[B8] Grotewold E. (2006). The genetics and biochemistry of floral pigments. Annu. Rev. Plant Biol. 57, 761–780. doi: 10.1146/annurev.arplant.57.032905.105248. PMID: 16669781

[B9] Hernández-Verdeja T. (2026). Regulation of chloroplast biogenesis and differentiation. J. Exp. Bot. 77, 1568–1581. doi: 10.1093/jxb/eraf530. PMID: 41351600 PMC13017677

[B10] Kobayashi K. Baba S. Obayashi T. Sato M. Toyooka K. Keränen M. . (2012). Regulation of root greening by light and auxin/cytokinin signaling in Arabidopsis. Plant Cell 24, 1081–1095. doi: 10.1105/tpc.111.092254. PMID: 22415275 PMC3336121

[B11] Sato Y. Morita R. Nishimura M. Yamaguchi H. Kusaba M. (2007). Mendel's green cotyledon gene encodes a positive regulator of the chlorophyll-degrading pathway. PNAS 104, 14169–14174. doi: 10.1073/pnas.0705521104. PMID: 17709752 PMC1955798

[B12] Susila H. Nasim Z. Gawarecka K. Jung J. Y. Jin S. Youn G. . (2023). Chloroplasts prevent precocious flowering through a GOLDEN2-LIKE-B-BOX DOMAIN PROTEIN module. Plant Commun. 4, 100515. doi: 10.1016/j.xplc.2023.100515. PMID: 36597356 PMC10203396

[B13] Tanaka Y. Sasaki N. Ohmiya A. (2008). Biosynthesis of plant pigments: anthocyanins, betalains and carotenoids. Plant J: For. Cell. Mol. Biol. 54, 733–749. doi: 10.1111/j.1365-313X.2008.03447.x. PMID: 18476875

[B14] van der Kooi C. J. Kevan P. G. Koski M. H. (2019). The thermal ecology of flowers. Ann. Bot. 124, 343–353. doi: 10.1093/aob/mcz073. PMID: 31206146 PMC6798827

[B15] Waters M. T. Wang P. Korkaric M. Capper R. G. Saunders N. J. Langdale J. A. (2009). GLK transcription factors coordinate expression of the photosynthetic apparatus in Arabidopsis. Plant Cell 21, 1109–1128. doi: 10.1105/tpc.108.065250. PMID: 19376934 PMC2685620

[B16] Wei Y. Yang H. Wang Y. Shen H. Zhang S. Yang Z. . (2025). PGA37 overexpression promotes chloroplast development in Arabidopsis roots through direct transcriptional activation of GLK2, ARR13, and ARR21. Plants (Basel Switzerland) 14 (9), 1270. doi: 10.3390/plants14091270. PMID: 40364299 PMC12073186

[B17] Zubo Y. O. Blakley I. C. Franco-Zorrilla J. M. Yamburenko M. V. Solano R. Kieber J. J. . (2018). Coordination of chloroplast development through the action of the GNC and GLK transcription factor families. Plant Physiol. 178, 130–147. doi: 10.1104/pp.18.00414. PMID: 30002259 PMC6130010

