# Supplementary figures and images for: Cytokinin-GLK regulatory module promotes the acquisition of photosynthetic activity and cell cycle re-entry in a green-flower mutant of Narcissus tazetta var. chinensis
Source: Front Plant Sci. 2026 May 20;17:1831530. doi: 10.3389/fpls.2026.1831530 (PMC13229977; doi:10.3389/fpls.2026.1831530)

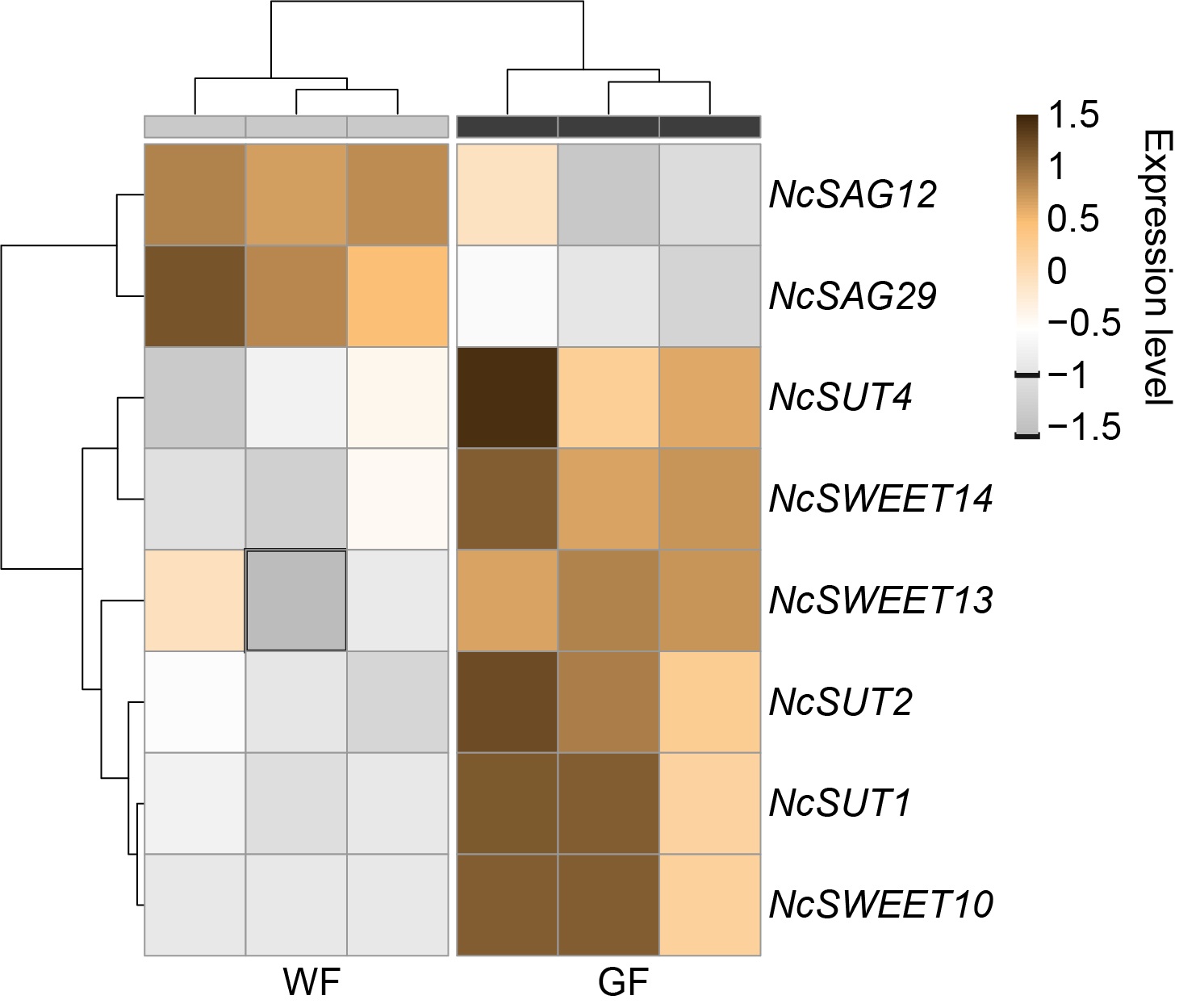

Supplement: Supplementary Figure 1 — Expression patterns of sugar transport and senescence-associated genes in WF and GF. The heatmap displays the differential expression of sucrose transporter genes (NcSUTs), sugar transporter genes (NcSWEETs), and senescence-associated genes (NcSAGs) between WF and GF samples. The color scale ranging from gray (−1.5) to brown (1.5) represents the Z-score normalized relative expression levels of the genes. Each column represents a biological replicate (n = 3). [file Image1.jpeg]

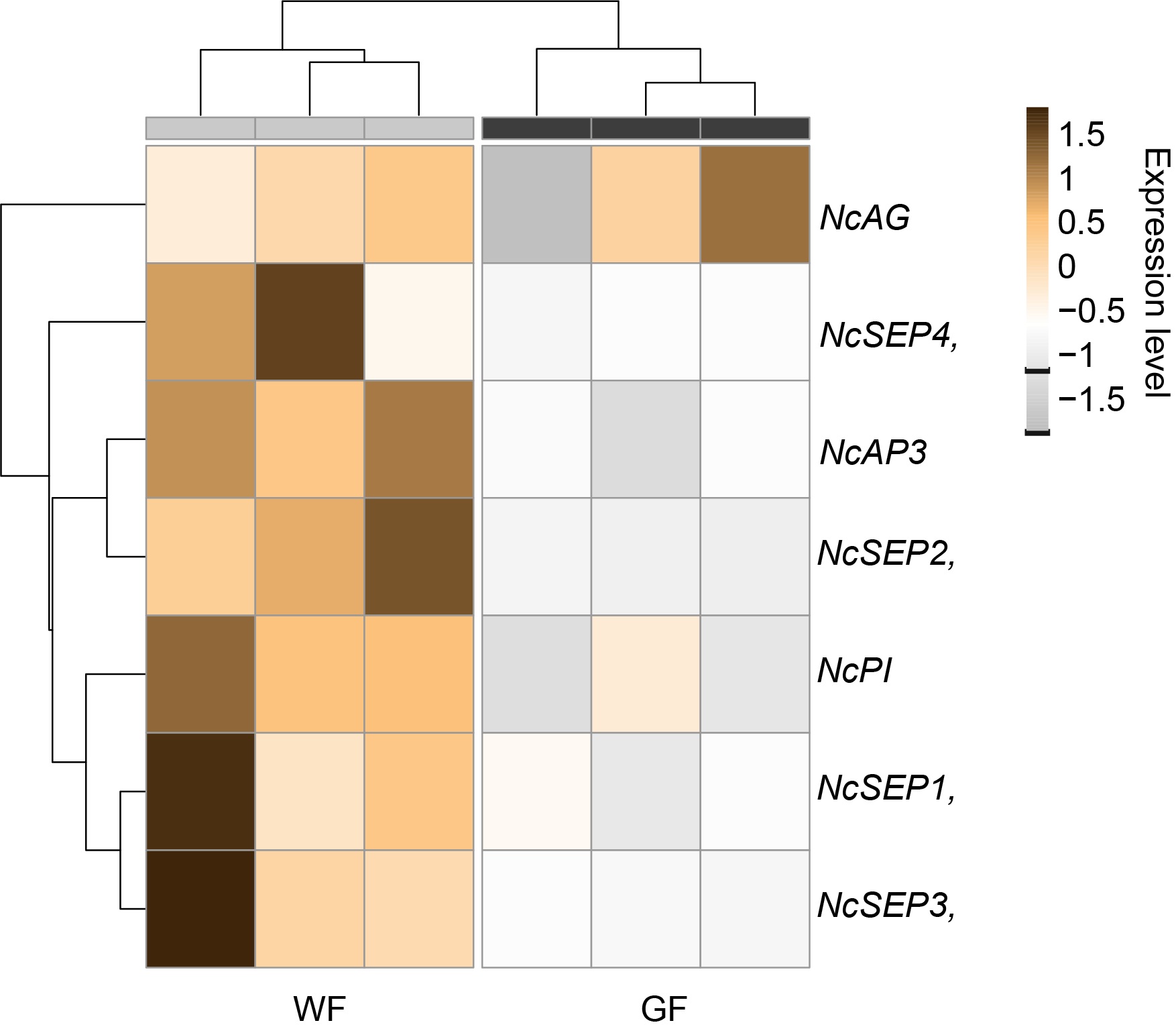

Supplement: Supplementary Figure 2 — Expression patterns of floral organ identity genes in WF and GF. The heatmap illustrates the expression profiles of MADS-box family genes involved in floral organ development (including NcAP3, NcPI, NcSEP1/2/3/4, and NcAG) in WF and GF samples. The color scale from gray (−1.5) to brown (1.5) indicates the Z-score normalized relative expression levels. Each column represents a biological replicate (n = 3). [file Image2.jpeg]

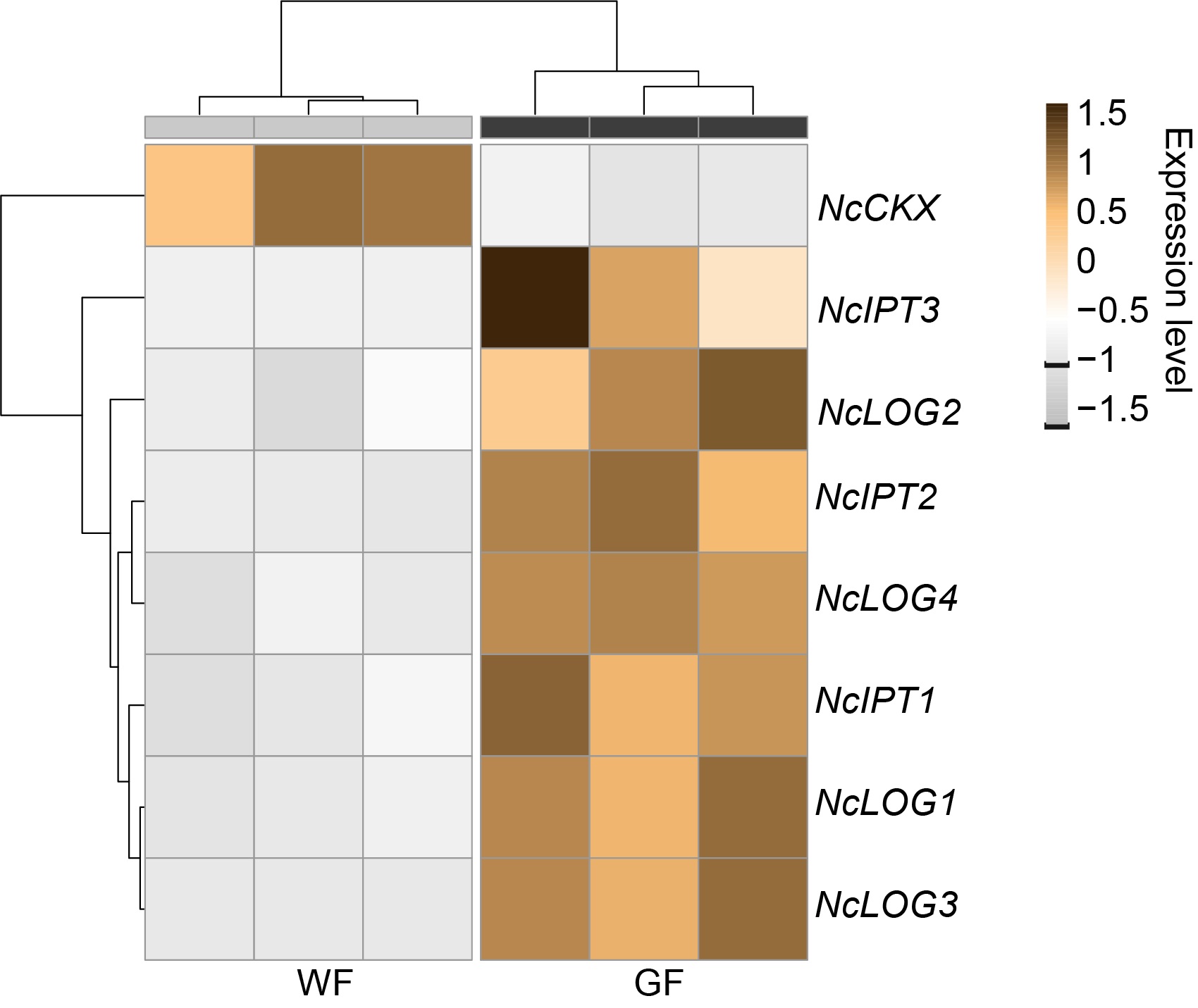

Supplement: Supplementary Figure 3 — Expression patterns of cytokinin metabolism-related genes in WF and GF. The heatmap analyzes the expression levels of genes involved in cytokinin biosynthesis (NcIPTs, NcLOGs) and degradation (NcCKX) in WF and GF. Biosynthesis-related genes are highly expressed in GF, whereas the degradation gene is highly expressed in WF. The color scale represents normalized expression levels (gray for low expression, brown for high expression). Each column represents a biological replicate (n = 3). [file Image3.jpeg]

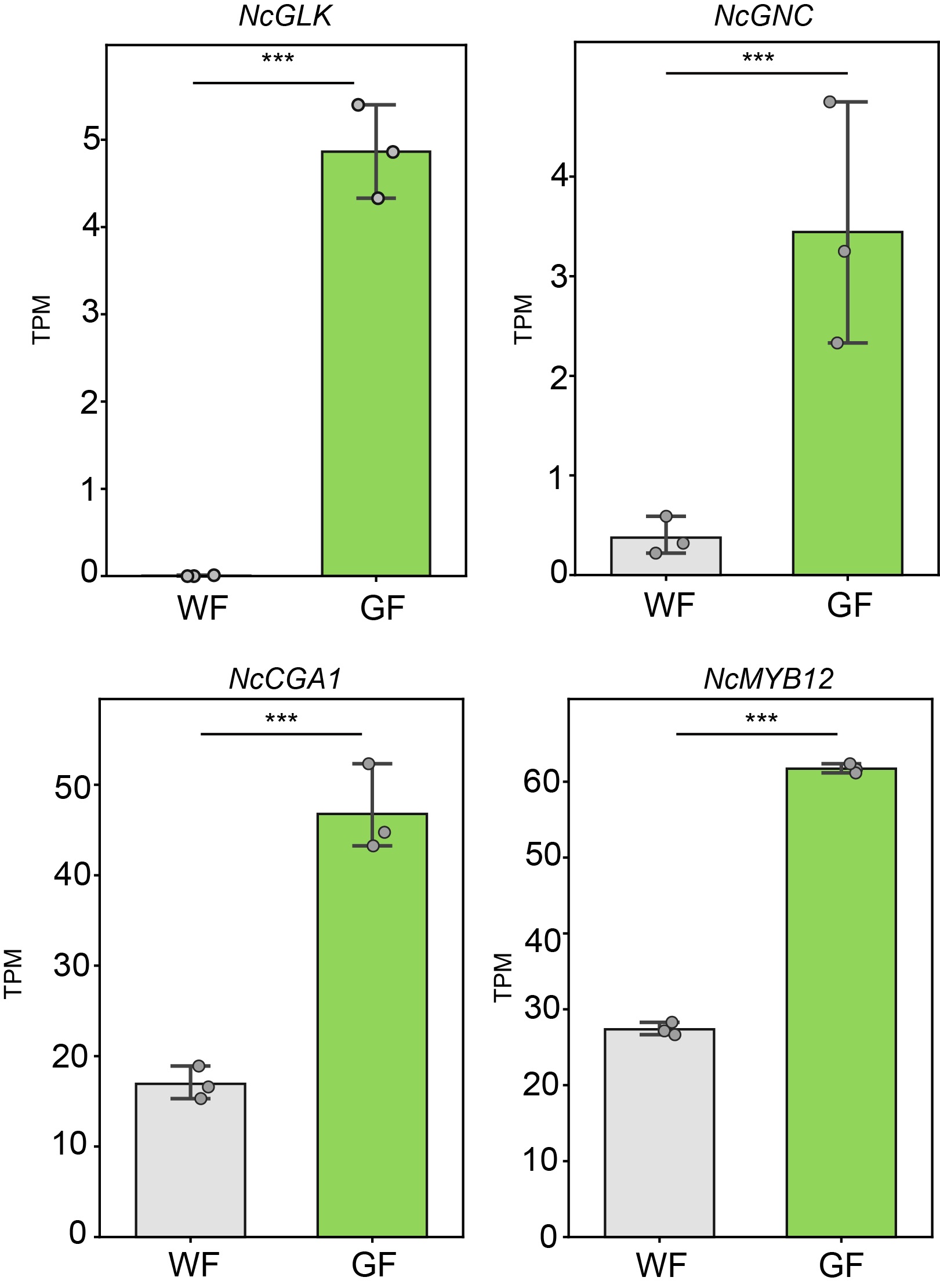

Supplement: Supplementary Figure 4 — Expression analysis of transcription factors related to chloroplast development and photosynthesis in WF and GF. Bar charts show the transcript abundance (represented by TPM values) of four transcription factors: NcGLK, NcGNC, NcCGA1, and NcMYB12 in WF and GF. Error bars represent the standard deviation of three biological replicates (Mean ± SD), and circles represent individual data points. Asterisks indicate high statistical significance between the groups (Student’s t-test, ***p < 0.001). [file Image4.jpeg]

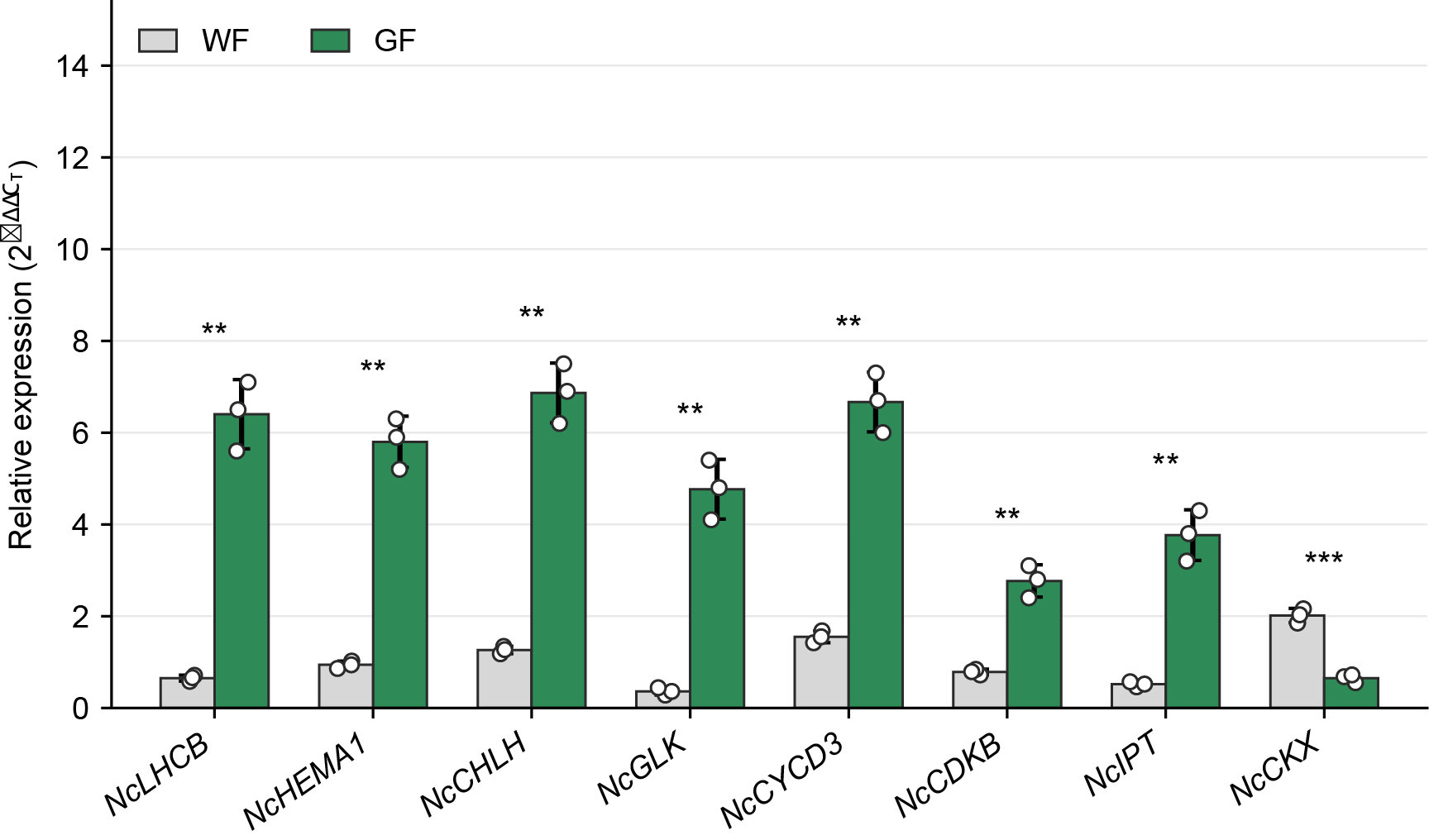

Supplement: Supplementary Figure 5 — Relative expression levels of representative genes involved in photosynthesis/chlorophyll biosynthesis (NcLHCB, NcHEMA1, and NcCHLH), the GLK regulatory module (NcGLK), cell-cycle progression (NcCYCD3 and NcCDKB), and cytokinin metabolism (NcIPT and NcCKX) in wild-type flowers (WF) and green-flower mutant tissues (GF). Expression levels were calculated using the 2−ΔΔCT method and normalized to WF. Bars represent means ± SD of three biological replicates, and individual points indicate replicate values. Statistical significance was determined using a two-tailed Student’s t-test; **P < 0.01, ***P < 0.001. [file Image5.jpeg]
